# Performance and characteristics of the Newborn Hearing Screening Program in Campania region (Italy) between 2013 and 2019

**DOI:** 10.1007/s00405-021-06748-y

**Published:** 2021-03-25

**Authors:** Rita Malesci, Valeria Del Vecchio, Dario Bruzzese, Ernesto Burattini, Gennaro Auletta, Monica Errichiello, Anna Rita Fetoni, Annamaria Franzè, Carla Laria, Fabiana Toscano, Antonio Caso, Elio Marciano

**Affiliations:** 1 Unit of Audiology, Department of Neurosciences, Reproductives and Odontostomatologic Sciences, University of Naples “Federico II”, via Pansini 5, 80131 Napoli, Italy; 2 Department of Public Health, University of Naples “Federico II”, Naples, Italy; 3 Otorhinolaryngology, Fondazione Policlinico Universitario A. Gemelli IRCCS, Department of Head and Neck Surgery Otorhinlaryngology, Catholic University of the Sacred Heart, Rome, Italy

**Keywords:** Newborn hearing screening, Coverage, Refer rate, Permanent childhood hearing loss, Positive predictive value, Risk factor

## Abstract

**Purpose:**

Universal newborn hearing screening (UNHS) in the first month of life is crucial for facilitating both early hearing detection and intervention (EHDI) of significant permanent hearing impairment (PHI). In Campania region, UNHS has been introduced in 2003 by the Regional Council Resolution and started on January 2007. The aim of this paper is to update a previous article describing the performance of the program since its implementation in the period between 2013 and 2019.

**Methods:**

A longitudinal retrospective study was carried at the Regional Reference Center III on 350,178 babies born in the analysis period. The paper reports the main results of overall coverage, referral rate, lost-to-follow-up rate,yield for PHI and shall determine various risk factor associations with hearing impairment

**Results:**

In Campania region, 318,878 newborns were enrolled at I level, with a coverage rate of 91.06%, 301,818 (86.18%) Well Infant Nurseries (WIN) and 17,060 (5.35%) Neonatal Intensive Care Unit (NICU) babies. PHI was identified in 413 children, 288 (69.73%) bilaterally and 125 (30.26%) unilaterally. The overall cumulative incidence rate of PHI was 1.29 per 1000 live-born infants (95% CI 1.17–1.42) with a quite steady tendency during the whole study period.

**Conclusions:**

This study confirms the feasibility and effectiveness of UNHS in Campania region also in a setting with major socioeconomic and health organization restrictions.The program meets quality benchmarks to evaluate the progress of UNHS. Nowadays, it is possible to achieve an early diagnosis of all types of HL avoiding the consequences of hearing deprivation.

## Introduction

Universal newborn hearing screening (UNHS) in the first month of life is crucial for facilitating both early hearing detection and intervention (EHDI) of significant permanent hearing impairment (PHI).

The incidence of PHI in newborn babies is generally assumed to amount approximately to 0.5–1.5/1000, but it may increase up to 3.5–6/1000 for children in school age [1]. In absence of EHDI [1], the consequences of this condition may include significant delays both in language development and in academic achievement.

The goal of EHDI is the prompt management of these disorders with a view to minimizing hearing deprivation while maximally stimulating auditory development during the peak period for neural growth. Therefore, the Joint Committee on Infant Hearing (JCIH) strongly recommends that all neonates should undergo hearing screening tests within the first month of life and that diagnosis should be made by 3 months of age, so that treatment and interventions can start by 6 months of age [2]. In fact, children who are early diagnosed can benefit from the timely fitting of hearing aids or cochlear implants [3, 4]. In this way, outcomes seems to be improved by early rehabilitation in PHI without any delay of speech, language and cognitive development [1]. The detrimental consequences of hearing deprivation can be avoided, and PHI children have the opportunity to grow up with normal developmental index scores, both in terms of academic and socioeconomic progress as well as in their emotional and psychological integrity [5]. An appropriate early intervention allows PHI children to have the same central auditory pathways than healthy ones.

In 2017, the Italian Ministry of Health introduced UNHS among the Essential Levels of Assistance (ELA) [6]. Therefore, the UNHS was declared mandatory nationwide. Neverthless, its implementation is entrusted to regional health agencies throughout the country and it is under legislative definition in some regions and than adopted in some maternal units in absence of an integrated territorial network.

In Campania region, the third region in Italy and the biggest one in South Italy in terms of number of births, UNHS was introduced in 2003 by the Regional Council Resolution and it has been universaly performed in a three levels setting.

Accordingly to preliminary data on the program implementation of UNHS in our region since 2003 [7], herein we aimed (i) to assess the results of UNHS between January 2013 and December 2019 in term of overall coverage, referral rate, lost-to-follow-up rate, yield for PHI and (ii) to evaluate the impact of risk factors on the PHI.

## Materials and methods

A longitudinal retrospective analysis was performed by the Unit of Audiology and Vestibology of the Department of Neuroscience, Reproductive and Odontostomatologic Sciences of the University of Naples Federico II between January 2013 and December 2019. The analysis describes the results of UNHS program in Campania region. All children born during that period, who were screened in the hearing screening program, were included in the study.

On October 31st 2003, Campania Region (Italy) enacted the regional law No. 3130, about “Universal Newborn Hearing Screeningˮ, adopting an organizational model structured in three levels. The first level (Level I) consists of 56 birth centers in which there are about 55,000 births every year, and by 18 neonatal intensive care units (NICU). The second level (Level II) is composed by 15 corporate structures responsible for the confirmation of the diagnosis (departments of Audiology and Phoniatrics, Otolaryngology). The third level (Level III) is the Regional Reference Center (RRC), supervised by the Unit of Audiology and Vestibology of the Neuroscience Department of the University of Naples “Federico IIˮ. The latter is responsible for the final treatment as well as the rehabilitation for children with HL or deafness.

Different protocols were adopted to screen infants who were admitted to well infant nurseries (WIN) and those who were admitted to NICU for more than 48 h.

At Level I, WIN were screened via two stages using Transient Evoked Automated Otoacoustic Emissions (TEOAE) measurements: the first one was made in the course of the second or third day of life, while the second one wase made between 2 and 3 weeks of age if the result had failed in one or both ears. All newborns were also evaluated for the occourrence of audiological risk factors.

Thus TEOAE and Automated Auditory Brainstem Response (A-ABR) were reserved to NICU infants prior the discharge. Premature babies did the screening at the end of the 35th gestational week or later.

Infants who failed both tests, either bilaterally or unilaterally, were referred at the Level II to the nearest pediatric audiology service to perform a comprehensive audiology evaluation with clinical click-evoked Auditory Brainstem Response (ABR) and Distortion Product Otoacoustic Emissions (DPOAE). Moreover, infants at risk for delayed/progressive and acquired HL required audiological evaluations following the tabular reference of the 2007 Position Statement [8], even if they had passed TEOAE. In case of HI identification, a Level III multidisciplinary diagnostic work-up, together with appropriate management, were provided by the RRC, Audiology and Vestibology Unit of the Neuroscience Department of the University of Naples "Federico II”.

### Diagnostic assessment

During the period of investigation, the project combined two different types of tests: OAE and ABR.

Both TEOAE and A-ABR were performed at birth centres or pediatric audiology services in a quite room, while babies were sleeping or at the end of feeding, by specifically trained personnel. The device used was Accuscreen^R^ Madsen newborn hearing screener (Natus) which detects both TEOAE and A-ABR (both tests only in case of WIN with risk factors or NICU babies). It is an automated device whose output simply indicates the final response score (“pass” or “refer”). TEOAE test was executed placing the ear plugs in both ears, one ear at a time, and its evaluation was based on noise-weighted averaging counting of significant signal peaks; stimuli were non-linear click sequences at 35 dB nHL with a frequency range of 1.5–4.5 kHz. A-ABR test required both the ear plug in the ear and the montage of 3 electrodes with impedance kept ≤ 3000 dines. The active/positive electrode was placed to the forehead, the exploring/negative electrode on the homolateral mastoid and the massa/ground electrode on the cheek. The clicks of A-ABR were delivered at a fixed intensity of 35 dB nHL. The tester is not expected to set any parameter of the device for each test: after the initial calibration routine, the recording session starts automatically. The device stops the recording as soon as the default “pass” criteria are met or after a given elapsed time. In the latter case, the response is scored as “refer”. During the recording session, the tester can decide to repeat the recording on the basis of qualitative information provided by the device about the stimulus stability and artifacts.

The diagnostic ABR evaluation with threshold identification was performed by an audiometrist with a specific expertise in this field in one of the pediatric audiology services, in a sound proof and faradized room, during spontaneous sleep. The device used was Neuro-Audio, Inventis. The test was performed by standard skin preparation and three electrodes montage with impedance kept ≤ 3000 dines. One active electrode was applied on the forehead, one exploring electrode was placed on the homolateral mastoid and one was a contralateral mass electrode. The standard procedure consists of alternate clicks at 21 pps, duration 0.1 ms, filter settings 100–2000 Hz and analysis time 12 ms. The protocol starts with a monaural stimulation at 80 dB HL for the identification of the three main waves I, III and V—for the determinations of peak and inter-peak latencies. After this step, the stimulus is decreased at 10-dB steps up to a minimum of 20 dB HL. Normal hearing was defined on the basis of presence and persistence of V wave, for acoustic stimuli < 30 dB nHL and HL was defined as presence and persistence of V wave for acoustic stimuli ≥ 30 dB nHL. Moreover, DPOAE (Neuro-Audio, Inventis; f2:f1 1.22, L2/L1 55/65 dB SPL) and tympanometry with 226- and 1000 Hz-tone probes (R36M, Resonance) were performed for each child to confirm the diagnosis of neurosensorial HL.

In this paper, the categorization of HL is based on the Bureau International for Audiophonology (Biap) classification [9] and includes: normal (< 20 dB HL), mild (21–40 dB HL), moderate (41–70 dB HL), severe (71–90 dB HL) and profound (> 91 dB HL).

While many screening programs include babies with permanent moderate-profound HL in their target groups, our program aims to identify children with permanent mild-profound and unilateral HL in the target group.

The UNHS efficiency was analyzed according to quality criteria defined by the American Academy of Pediatrics in 2010, including referral rate ≤ 4%, false-positive rate ≤ 3%, compliance for follow-up testing 95%, adherence ≥ 70%.

### Data collection and measures

The analysis of data was allowed by RRC, but also by birth centers and NICU involved in UNHS of Campania region. The information of Level I was collected in birth centers while NICU and summary reports were transmitted every month to RRC. Instead, the data of Level II and III were collected by the clinical records at the RRC and contained details of PHI children: number of children tested and referred to Level III centers; number of children with unilateral/bilateral HL; degree of HL (mild/moderate and severe/profound), information on risk factors of babies included in the screening protocol, age at diagnosis. All these data were tracked in an internal database. Risk factors included in the study were as follows: part of the JCIH list replacing the item “entry in NICU” with prematurity (< 37 weeks) and low birth weight (< 2500 g); documented in the literature as risk factors for HL but not part of the JCIH list.Fourtheen risk factors satisfied the selection criteria and were included (Table 1).

**Table 1:** Audiological risk factors

	Risk factor
1	Low birth weight and/or prematurity
2	Assisted ventilation (to aid with breathing for more than 5 days after delivery)
3	Birth asphyxia (Apgar score 0–6 at 5 min)
4	Severe hyperbilirubinemia
5	Hydrocephalus
6	Ototoxic medications (e.g. aminoglycosides, loop diuretics)
7	Stigmata or other findings associated with a known syndrome to include a sensorineural and/or conductive hearing loss
8	Family history of permanent childhood sensorineural hearing loss
9	Craniofacial anomalies including those with morphological abnormalities of the pinna and ear canal
10	In utero TORCH infection
11	Respiratory distress (presence of at least two of the following criteria: respiratory rate more than 60 per minute/subcostal or intercostal recession/expiratory grunt or groaning)
12	Meningitis and sepsis with positive CSF and blood cultures, respectively
13	Parental concern
14	Head trauma or intracranial hemorrhage

### Analysis of data

All statistical analyses have been conducted using the statistical platform R (R core team 2020). Variables were described using standard descriptive statistics; mean ± standard deviaton in case of numerical variables and frequencies with percentage in case of categorical variable.

Cumulative Incidence Rates were computed, for each calendar year, as the number of babies, who were born in that year, with a defined diagnosis of unilateral/bilateral HL divided by the number of babies born in that year who entered the first stage screening. The corresponding 95% Confidence Intervals (95% CI) were estimated using the normal aproximation.

Positive predictive values (PPVs) were defined as the percentage of screen positives that have the target condition.

The association between selected risk factors and HL was quantified by computing crude Odds Ratios (ORs) with the corresponding 95% CI.

This study was approved by University of Naples Federico II Ethics Committee (protocol number 56/18 on 14/02/2018). All procedures performed in studies involving human partecipants were in accordance with the ethical standards of the institutional and national research committee and with 1964 Helsinki declaration and its later amendments or comparable ethical standards. Our study was a national screening project, and there was no need to obtain informed consent from the subjects.

## Results

**Fig. 1:**
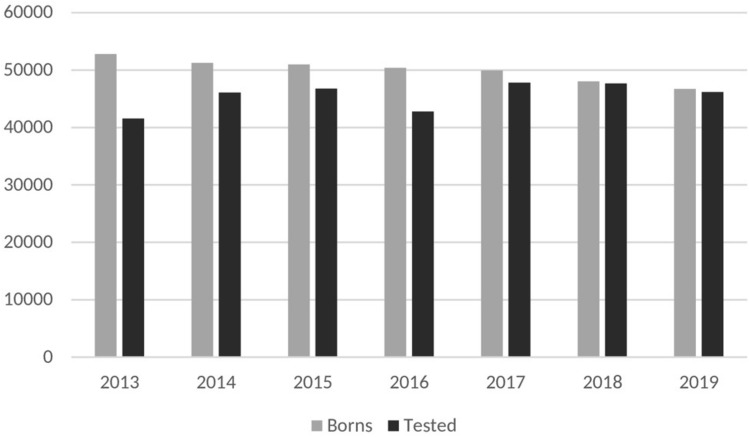
Coverage by year

A total of 350,178 babies were born in Campania region between January 2013 and December 2019. Level I screening was performed on 318,878 in 56 different birth centers. Overall coverage rate was 91.06%; howewer, coverage increased since 2017 reaching a rate of 98.8% by 2019. Figure 1 shows coverage rate by year from 2013 to 2019.

**Fig. 2:**
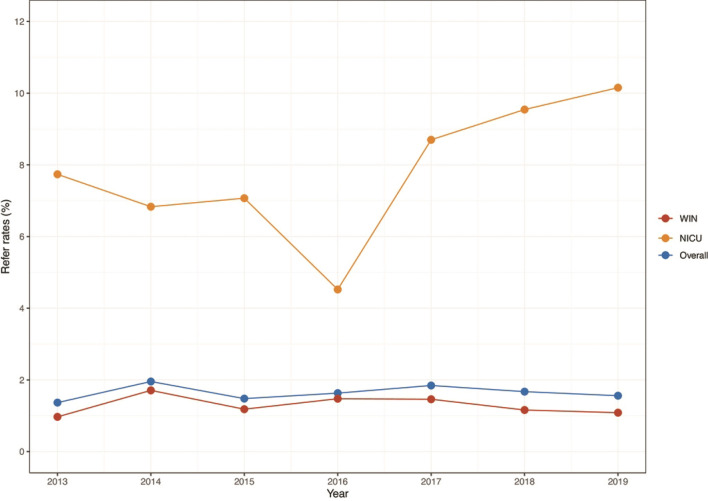
Screen refer rates for overall, NICU and well babies

Thus at Level I, 301,818 (94.64%) WIN and 17,060 (5.35%) NICU babies were tested. Among these, refer results were obtained in 5256 (1.65%) namely 3907 (74.03%) WIN and 1349 (25.66%) NICU babies corresponding to overall referral rate of 1.65%. Figure 2 shows the total refer rate for well babies and NICU babies by year of birth cohort. Notably, 4651 (1.45%) infants that passed at Level I presented at least one risk factor for HL.

Consequently, 9388 children (2.94% of all screened newborns) were enrolled in the Level II in one of the Audiology clinic partecipating in the project to perform ABR, 8774 (93.46%) of them were tested while 614 (6.54%) children were not rescreened due to parental refusal or because they were lost to follow-up. As result of Level II, 8107 (92.40%) newborns were discharged while 667 (7.60%) failed the test either bilaterally or unilaterally and were referred to the Level III center for the clinical diagnosis of HL. Level III evaluation was performed on 807, namely 472 (58.48%) WIN, 335 (41.51%) NICU. They included 667 (82.65%) children that failed Level II and 140 (17.34%) children who were identified by the audiological surveillance among those who had lost the screening test at birth. At the end of UNHS program PHI was identified in 413 (51.17%) babies. Unlikely 113 (14%) babies did not undergo ABR testing because their parents refused to continue or they were lost to follow-up. Data are summarized in Table 2.

**Table 2:** Third level screening results in overall, NICU babies and WIN

Type HL	Overall (%)	NICU (%)	WIN (%)
ANSD	18 (4.36)	7 (5.26)	11 (3.93)
Bilateral SNHL	254 (61.5)	67 (50.38)	187 (66.79)
Unilateral SNHL	100 (24.21)	41 (30.83)	59 (21.07)
Bilateral CHL	16 (3,87)	5 (3,76)	11 (3,93)
Unilateral CHL	23 (5.57)	13 (9.77)	10 (3.57)
Mixed HL	2 (0.48)	0 (0)	2 (0.71)
Mild	60 (14.53)	20 (15.04)	40 (14.29)
Moderate	167 (40.44)	60 (45.11)	107 (38.21)
Severe	23 (5.57)	9 (6.77)	14 (5)
Profound	163 (39.47)	44 (33.08)	119 (42.5)

**Fig. 3:**
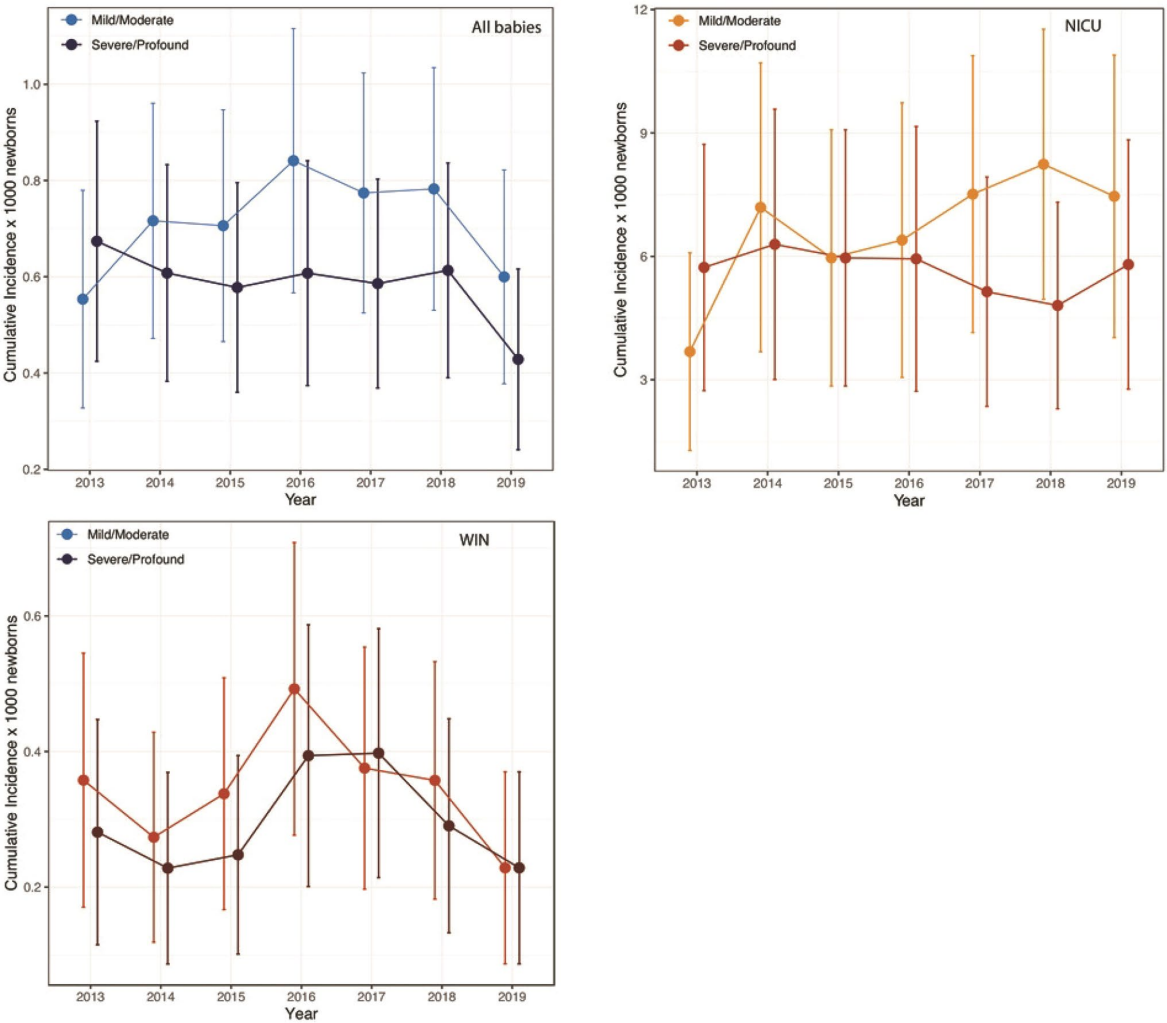
Cumulative incidence rates of HL stratified according to severity and birth center

The overall cumulative incidence rate of HL was 1.29 per 1000 live-born infants (95% CI 1.17–1.42) with a quite steady tendency during the whole study period (Fig. 3). The incidence of HL was significantly higher in the high-risk infants, 12.3 per 1000 (95% CI 10.6–14.0) than in low-risk babies, 0.67 per 1000 (95% CI 0.58–0.77). This difference was consistent during the surveyed years. Bilateral HL was identified in 288 babies (69.73%) and unilateral HL in 123 babies (29.78%). In addition, regarding the type, sensorineural HL (SNHL) was detected in 354 (85.71%), conducted HL (CHL) in 39 (9.44%), mixed HL in 2 (0.48%) and Auditory Neuropaty Spectrum Disorder (ANSD) in 18 (4.35%). Data are summarized in Table 2.

According to BIAP classification [9], the severity of HL resulted profound in 163 (39.46%), severe in 23 (5.56%), moderate in 167 (40.43%), mild in 60 (14.52%). Among WIN, the threshold was profound in 119 (42.40%), severe in 14 (5%), moderate in 107 (38.21%), mild in 40 (14.29%). Among NICU babies, it was profound in 44 (33.08%), severe in 9 (6.77%), moderate in 60 (45.11%), mild in 20 (15.04%). Data are summarized in Table 2.

**Fig. 4:**
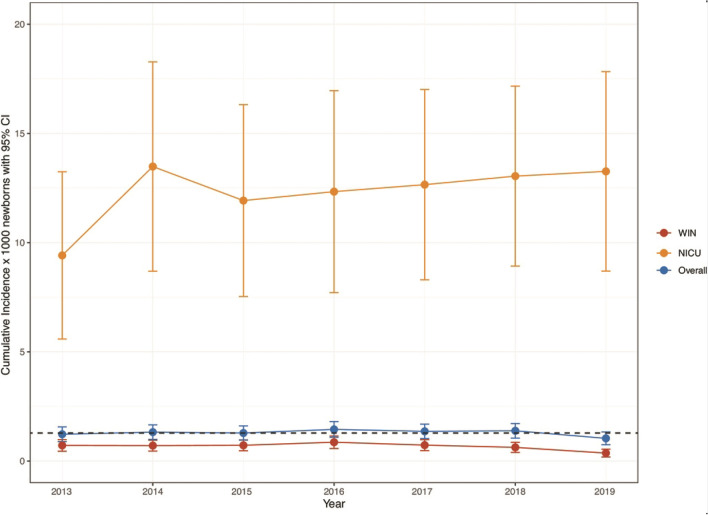
Cumulative incidence rate of hearing loss for overall, NICU and well babies in the period of study

The cumulative incidence rates of HL stratified according to severity (mild/moderate and severe/profound), and birth center are displayed in Fig. 4.

Table 3 shows a matrix of PPVs for all referrals (bilateral and unilateral referrals), for all PHL for three population groups (all babies, NICU and WIN). Values are calculated using data for all births between January 1st 2013 and December 31st 2019. As expected, PPVs were higher in NICU (15.6%; 95% CI 13.7–17.6) than in WIN (5.2%; 95% CI 4.5–5.9) due to the highest HL incidence observed in NICU.

**Table 3:** PPVs (95% CI) for all referrals, for all PHL in overall, NICU babies and WIN

	Tested	Refer	HL	PPV % (95% CI)
Overall	318,878	5256	413	7.9 (7.1–8.6)
WIN	301,818	3907	203	5.2 (4.5–5.9)
NICU	17,060	1349	210	15.6 (13.7–17.6)

Interestingly among the risk factors considered in the study, ototoxic drugs (0.62%) have the highest prevalence followed by respiratory distress (0.61%), prematurity (0.57%), severe hyperbilirubinemia requiring phototherapy (0.57%), mechanical ventilation (0.46%). Among babies with PHI, the more prevalent risk factors were family history (13.1%) followed by respiratory distress (11.9%), severe hyperbilirubinaemia (10.9%), ototoxic drugs (10.2%). Prevalence of risk factors in the NICU and WIN across each risk factor are reported in Table 4.

**Table 4:** Prevalence of risk factors in overall,WIN, NICU, PHI babies and odds ratios of risk factors for hearing loss

Risk factor	Overall	WIN	NICU	PHI	OR 95% CI
Craniofacial anomalies	126 (0.04)	63 (0.02)	63 (0.37)	25 (6.1)	249.61 (157.78–394.9)
Congenital infection	1161 (0.37)	945 (0.31)	216 (1.28)	13 (3.1)	9.39 (5.39–16.37)
Low birth weight < 1500	911 (0.29)	55 (0.02)	856 (5.26)	22 (5.3)	21.36 (13.83–32.99)
Head trauma or intracranial hemorrhage	100 (0.03)	10 (0)	90 (0.53)	3 (0.7)	25.54 (8.05–81.01)
Prematurity	1816 (0.57)	127 (0.04)	1689 (10.93)	10 (2.4)	4.55 (2.42–8.53)
Birth asphyxia	156 (0.05)	15 (0)	141 (0.83)	4 (1)	21.57 (7.95–58.53)
Respiratory distress	1933 (0.61)	127 (0.04)	1806 (11.78)	49 (11.9)	23.92 (17.69–32.35)
Ototoxic drugs	1970 (0.62)	247 (0.08)	1723 (11.44)	42 (10.2)	19.58 (14.18–27.02)
Severe hyperbilirubinemia	1798 (0.57)	604 (0.2)	1194 (7.49)	45 (10.9)	23.58 (17.24–32.26)
Sepsis	654 (0.21)	59 (0.02)	595 (3.6)	20 (4.8)	26.77 (16.96–42.24)
Mechanical ventilation	1474 (0.46)	93 (0.03)	1381 (8.77)	29 (7)	17.33 (11.84–25.36)
Hydrocephalus	10 (0)	1 (0)	9 (0.05)	1 (0.2)	96.4 (12.03–772.53)
Family history	754 (0.24)	446 (0.15)	308 (1.83)	54 (13.1)	73.84 (54.9–99.3)
Respiratory distress	153 (0.05)	60 (0.02)	93 (0.55)	5 (1.2)	27.6 (11.25–67.71)
Syndromes	49 (0.02)	20 (0.01)	29 (0.17)	36 (8.7)	3792 (1751–8213)

All risk factors emerged as significant predictors of HL with the highest impact of syndromes (OR 3792; 95% CI 1751–8213) and the lowest of prematurity (OR 4.55; 95% CI 2.42–8.53). The full listing of risk factors with their distribution stratified by presence/absence of HL is reported in Table 4.

The mean age at hearing loss diagnosis was 5.04 ± 3.31 months: 4.50 ± 3.06 months in WIN babies and 6.09 ± 3.53 months in NICU babies.

## Discussion

UNHS allows the EHDI to increase the possibility of appropriate speech and language development and to reduce neurodevelopmental problems of PHI.

UNHS program has became standard of care in many countries around the world. The feasibility and efficiency of these experiences vary depending on the level of healthcare particularly in developing countries [10].

In the last decade, UNHS programs were implemented in Italy through polices at a local or regional level with a progressive diffusion of screening coverage.

To the best of our knowledge, our study is the largest report on the UNHS program in a national representive sample of Italian births. Previous reports provided results of smaller cohorts of same Regions [11–13] or showed the experience of single hospital [14–20]. Indeed, in our knowledge, it shows the highest sample among Italian reports in literature. In fact, Campania region is one of the most populated areas of Southern Italy. It counts about 5.767.467 inhabitans [21] divided into five provinces (Napoli, Salerno, Avellino, Benevento, Caserta). Approximately, 60,000 infants are born every year and its birth rate is one of the most elevate in the country.

Overall, our study provides data on 318,878 of 350,178 newborns (91.06%) that underwent to UNHS in Campania region over seven years of UNHS program. The overall referral rate was 1.65% (1.29% in WIN, 7.91% in NICU) and PPV was 7.9% (5.2% inWIN, 15.6% in NICU). Referral rate was comparable to what reported in litterature and the observed PPVs were also consistent with data obtained in analogous setting [22].

The lost to follow-up (LTF) rate, that is a common factor affecting the screening procedures,defined as percentage babies who did not receive or complete the definitive diagnosis, was 20.14% (6.14% at Level II and 14% at Level III).

All effort was found to improve regional coverage rates compared to our previous report in 2013 [7], although the regional coverage rate remains a bias of UNHS in Campania Region.The major reason is that about 50 percent of maternity units are private health services, whose engagement in the activities of the National Health Service are still challenging. Another issue could be the incomplete data collection by Level I facilities due to the absence of a functioning reporting network between these and the Level III. Neverthless, this critical issue will be overcome by implementation of a portal web connecting audiological services, including information on demographic and audiological data for clinical and statistical evaluations. However, the coverage rate has constantely increased over the years 2017–2019 up to 95% showing a wide diffusion of hearing screening program in Campania region, and a better reporting of coverage by monitoring activity of the Level I (95.69%) in 2017, 98.18% in 2018 e 99.80% nel 2019).

The LTF rate is a common problem reported from all the countries where screening programs have been established [23–26]. In our study, this rate still needs to be improved but it is already better than the value of 40% reported by an American review [27].

This problem was discussed in the periodical meetings with the professionals of the provincial referral services taking part in the project. Part of the training for the next years will be dedicated to improve tracking techniques for children who are referred from the WIN and the NICU.

Interestingly, the overall incidence of congenital HL was 1.29/1000. This result is close to estimates in European studies that it is 1.12/1000 and increases with the age to 1.33/1000 in acquired and delayed on set HL [28] and to that of other Italian studies [17, 19, 20, 29, 30]. The incidence in babies admitted to NICU is more elevated than WIN: 12.3/1000, almost 20 times higher than WIN (0.67/1000). This finding is consistent with the increased prevalence of HL in studies on other high-risk populations [31].

Interestingly, this study provides evidence for detection of mild HL unlike other UNHS programs that aim to identify all children with a moderate-profound HL in the better hearing ear. The cumulative incidence rate of mild HL was relevant both in in WIN (14.29%) and in NICU babies (15.04%). The early detection of mild HL is particularly relevant because of the negative impact on the linguistic and curricular outcomes of these kind of HL [32].

A major aim of this study was to evaluate the risk factors associated with congenital HL. It is widely acknowledged that children with congenital or neonatal risk factors need to be tested during the neonatal period and to be closely monitored for late-onset HL.

Our study was mainly favored by the relatively large sample size that allows a full assessment of several risk factors.

Moreover, the leading RF in babies with HL resulted family history (13.1%) followed by respiratory distress (11.9%), severe hyperbilirubinaemia (10,9%) and ototoxic drugs (10.2%). The prevalence of the majority of the risk factors in children with HL was considerably higher than in the general population.

Many risk factors were significantly associated to HL albeit with a different relevance. The most relevant risk factors were syndromes, craniofacial anomalies, exposure to ototoxic drugs for > 5 days, hydrocephalus and familiarity.

Genetic causes account for at least 50–60% of childhood HL in developed countries and can be classified according to the pattern of inheritance, to the presence (syndromic) or absence (non syndromic) of distinctive clinical features, or to the identification of the causal mutation. Syndromes associated with HL, such as Waardenburg, Pendred, Down, Usher syndromes were frequently identified in accordance with literature [33–35].

Craniofacial abnormalities (including microtia, atresia, ear dysplasia, oral clefting) were more frequently found in hearing impaired newborns than in those who passed the screening. These findings correspond with the results of similar studies in which craniofacial anomalies were described as an independent risk factor for HL [36].

HL is a manifestation of the long-term complications in newborns with hydrocephalus [37]. The most common causes of pediatric hydrocephalus in children are brain bleeds as a result of prematurity, spina bifida, brain tumors, infection, and head injury. In our study, out of ten newborns identified only one had unilateral SNHL which was associated with low weight at birth, posthemorrhagic hydrocephalus and brainstem symptoms at the time of diagnosis of hydrocephalus. Howewer, the very large witdh of the corresponding 95% CI must impose caution in the interpretation of its impact on HL.

Ototoxic drugs are prescribed to babies to treat serious infections or birth complications. There is a growing concern that the administration of aminoglycoside treatment in the noisy envviroment of the NICU may lead to hearing impairment as well as to association of other clinical conditions [38]. HL resulting from the use of these antibiotics may also have a genetic component [39].

Our data confirm that positive family history is a risk factor for early, progressive or delayed onset PHI as well documented in literature [40]. Moreover, JCIH 2019 supports recommendations for audiologic diagnostic follow-up in children who pass newborn hearing screening based on etiology of family HL [24–26].

According to the actual raccomendations [2], UNHS in Campania Region permits to confirm promptly the diagnosis in newborns at 5.04 months ± 3.31:4.50 months ± 3.06 in WIN babies and 6.09 months ± 3.53 months in NICU babies. The infants at high risk require more control and this explain an increased time for obtaining a definitive diagnosis. However, it is in time to have all benefits from an early habilitation. Before the advent of UNHS, its introduction and implementation, the average age to diagnose bilateral moderate-profound HL was 26 months and habilitation with hearing aids was started at 32.2 months. The earlier identification of HL through UNHS provide earlier access to intervention and improve developmental outcomes of children with congenital HLs. Further it is now evident that children who receive earlier intervention have better language scores at 5 years of age [41].

## Conclusions

This study confirms the feasibility and effectiveness of UNHS in Campania region also in a setting with major socioeconomic and health organization restrictions. There was a continuous improvement in the performance of the screening program over the last seven years. The program meets quality benchmarks to evaluate the progress of UNHS program according to its three pillars of universality, timely detection, and overreferral. Nowadays, it is possible to achieve an early diagnosis of all types of HL avoiding the consequences of hearing deprivation. It permits to focus on habilitation and development of the child instead that on the rehabilitation, as in the past.

## Abbreviations

UNHS: Universal newborn hearing screening
EHDI: Early hearing detection and intervention
PHI: Permanent hearing impairments
WIN: Well infant nurseries
NICU: Neonatal Intensive Care Unit
JCIH: Joint Committee on Infant Hearing
HL: Hearing loss
Level I: First level
Level II: Second level
Level III: Third level
RRC: Regional Reference Center
TEOAE: Transient evoked otoacoustic emission
A-ABR: Automated auditory brainstem response
PPVs: Positive predictive values
95% CI: 95% Confidence intervals
ORs: Odds ratios
LTF: Lost to follow-up
ANSD: Auditory neuropathy spectrum disorder
